# Pathology-confirmed versus non pathology-confirmed cancer diagnoses: incidence, participant characteristics, and survival

**DOI:** 10.1007/s10654-019-00592-5

**Published:** 2019-12-20

**Authors:** Kimberly D. van der Willik, Liliana P. Rojas-Saunero, Jeremy A. Labrecque, M. Arfan Ikram, Sanne B. Schagen, Bruno H. Stricker, Rikje Ruiter

**Affiliations:** 1 Department of Psychosocial Research and Epidemiology, Netherlands Cancer Institute, Plesmanlaan 121, 1066 CX Amsterdam, The Netherlands; 2 Department of Epidemiology, Erasmus MC - University Medical Center Rotterdam, PO Box 2040, 3000 CA Rotterdam, The Netherlands; 3 Brain and Cognition, Department of Psychology, University of Amsterdam, Nieuwe Achtergracht 129B, 1018 WT Amsterdam, The Netherlands

**Keywords:** Cancer diagnosis, Pathology, Epidemiology, Cohort study, Survival

## Abstract

Cancer diagnoses which are not confirmed by pathology are often under-registered in cancer registries compared to pathology-confirmed diagnoses. It is unknown how many patients have a non pathology-confirmed cancer diagnosis, and whether their characteristics and survival differ from patients with a pathology-confirmed diagnosis. Participants from the prospective population-based Rotterdam Study were followed between 1989 and 2013 for the diagnosis of cancer. Cancer diagnoses were classified into pathology-confirmed versus non pathology-confirmed (i.e., based on imaging or tumour markers). We compared participant characteristics and the distribution of cancers at different sites. Furthermore, we investigated differences in overall survival using survival curves adjusted for age and sex. During a median (interquartile range) follow-up of 10.7 (6.3–15.9) years, 2698 out of 14,024 participants were diagnosed with cancer, of which 316 diagnoses (11.7%) were non pathology-confirmed. Participants with non pathology-confirmed diagnoses were older, more often women, and had a lower education. Most frequently non pathology-confirmed cancer sites included central nervous system (66.7%), hepato-pancreato-biliary (44.5%), and unknown primary origin (31.2%). Survival of participants with non pathology-confirmed diagnoses after 1 year was lower compared to survival of participants with pathology-confirmed diagnoses (32.6% vs. 63.4%; risk difference of 30.8% [95% CI 25.2%; 36.2%]). Pathological confirmation of cancer is related to participant characteristics and cancer site. Furthermore, participants with non pathology-confirmed diagnoses have worse survival than participants with pathology-confirmed diagnoses. Missing data on non pathology-confirmed diagnoses may result in underestimation of cancer incidence and in an overestimation of survival in cancer registries, and may introduce bias in aetiological research.

**Electronic supplementary material:**

The online version of this article (10.1007/s10654-019-00592-5) contains supplementary material, which is available to authorized users.

## Background

With ageing populations worldwide, the incidence of cancer is rising. In 2018, 17 million people were diagnosed with cancer and 9.6 million people died from cancer [1]. Accurate and complete registration of incident cancers is pivotal for cancer statistics. However, most cancer registries primarily rely on pathology databases. Although this limits the risk of false-positive diagnoses, it may result in under-registration of cancers that are diagnosed purely on the basis of other sources than pathology, such as imaging features or tumour markers [2, 3]. This may lead to an underestimation of cancer incidence and to inaccurate estimates of survival. Furthermore, aetiological studies often only include patients with a pathology-confirmed cancer diagnosis, which may induce bias if pathological confirmation is related to patient or cancer characteristics.

Several studies have investigated characteristics of patients with unstaged cancer based on the Surveillance, Epidemiology and End Results (SEER) database [4–6] or state cancer registries in the United States [7–9]. It was found that unstaged cancer occurs more often in patients with older age and in patients residing in nursing homes. Furthermore, unstaged cancers were often cancers with a poor survival such as oesophagus-, liver-, and pancreatic cancer [6, 10]. Missing cancer stage was explained by different reasons such as failure of the registry system, refusal for diagnostic testing, or absence of therapeutic consequences of staging. However, tumour grade was known in the majority of the unstaged cancers, which suggests that the studied cancer population is a combination of patients with missing cancer stage, but with pathological confirmation of the cancer, and patients with missing both cancer stage and pathology. Therefore, the incident number of patients with a cancer diagnosis based on other sources than pathology and their characteristics remain largely unknown.

Patients with suspected cancer undergo an extensive diagnostic work-up that includes physical examination, laboratory assessments, imaging features, and pathology. In some patients, pathology is not included in the diagnostic work-up of cancer. In this study, we will refer to these cancer diagnoses as ‘non pathology-confirmed’ diagnoses. If pathology is used to confirm the cancer diagnosis, we will use the term ‘pathology-confirmed’ diagnosis.

We hypothesized that pathology is more often omitted in older, vulnerable patients with impaired survival. Insight into the number of non pathology-confirmed cancer diagnoses and identification of the reasons for omitting pathology in the diagnostic work-up of cancer could stimulate and facilitate cancer registries and aetiological research studies to capture these cancer diagnoses. In the current study, we therefore investigated the number of participants with a non pathology-confirmed cancer diagnosis, their characteristics, and the overall and cancer-specific survival in the population-based Rotterdam Study.

## Methods

### Study population

This study is embedded within the Rotterdam Study, a prospective population-based cohort designed to study the occurrence and determinants of age-related diseases in the general population. The objectives and design have been described in detail previously [11]. In 1989, all residents aged ≥ 55 years of the district Ommoord in Rotterdam, the Netherlands, were invited to participate. This initial cohort comprised 7983 participants (response of 78%) and was extended with a second subcohort in 2000 with 3011 participants (response of 67%) who had become 55 years of age or moved into the study district. In 2006, the cohort was further extended with 3932 participants (response of 65%) aged ≥ 45 years. In total, the Rotterdam Study comprises 14,926 participants aged ≥ 45 years. The current study includes all participants who provided informed consent for follow-up data collection without a history of cancer at study entry (N = 14,024).

The Rotterdam Study has been approved by the Medical Ethics Committee of the Erasmus MC (registration number MEC 02.1015) and by the Dutch Ministry of Health, Welfare and Sport (Population Screening Act WBO, license number 1071272-159521-PG). The Rotterdam Study has been entered into the Netherlands National Trial Register (NTR; www.trialregister.nl) and into the WHO International Clinical Trials Registry Platform (ICTRP; www.who.int/ictrp/network/primary/en/) under shared catalogue number NTR6831. All participants provided written informed consent to participate in the study and to have their information obtained from treating physicians.

### Assessment of incident cancer

Diagnosis of incident cancer was based on medical records of general practitioners (including hospital discharge letters) and furthermore through linkage with Dutch Hospital Data (Landelijke Basisregistratie Ziekenhuiszorg), histology and cytopathology registries in the region (PALGA), and the Netherlands Cancer Registry. Using different sources of cancer diagnoses, the Rotterdam Study aims to capture also the non pathology-confirmed diagnoses. Incident cancer was defined as any primary malignant tumour, excluding non-melanoma skin cancer. Each primary malignant tumour was registered, so that participants could have been diagnosed with multiple cancers. Cancer diagnoses were coded independently by two physicians and classified according to the International Classification of Diseases, 10th revision (ICD-10). In case of discrepancy, consensus was sought through consultation with a physician specialised in internal medicine. Level of uncertainty of diagnosis was established as: certain (pathology-confirmed), probable (e.g., based on imaging features or elevated tumour markers without pathological confirmation), and possible (e.g., based on symptoms and physical examination, without further analysis and without pathological confirmation). Date of diagnosis was based on date of biopsy (solid tumours), laboratory assessment (haematological tumours), or—if unavailable—date of hospital admission or hospital discharge letter. For non pathology-confirmed cancers, we used the date of imaging, date of laboratory assessment, date of physical examination, or—if unavailable—the date of hospital admission or hospital discharge letter. Follow-up was completed up to January 1st, 2014. In case of multiple cancers within one participant, we only included the first diagnosis for analyses.

### Assessment of mortality

Information on vital status was updated continuously. Date of death was obtained and verified through notification by the municipal administration. Cause of death was obtained through follow-up of records of general practitioners and hospital discharge letters, and was classified according to the ICD-10 by two research physicians independently. Thereafter, a medical expert in the field reviewed all coded events. Cancer-specific mortality was defined as mortality attributed to malignant neoplasms (ICD-10 C00-C97).

### Assessment of characteristics

During home interviews at study entry, participants provided information on marital status, educational level, smoking status, and alcohol use. Marital status was categorised as living with or without partner. Educational level was classified into primary education, lower education (lower/intermediate general education or lower vocational education), intermediate (intermediate vocational education or higher general education), or higher (higher vocational education or university). Smoking habits were categorised as never, current, or former smoker. Alcohol use was classified into any use or no use of alcohol. At the research centre, height and weight were measured from which the body mass index (BMI; kg/m^2^) was computed. Hypertension was defined as a resting blood pressure exceeding 140/90 mmHg or the use of blood pressure lowering medication [12]. Diabetes was defined as use of antidiabetic medication, fasting serum glucose level ≥ 7.1 mmol/L, or random serum glucose level ≥ 11.1 mmol/L [13]. History of stroke, coronary heart disease (myocardial infarction, percutaneous coronary intervention, or coronary artery bypass grafting), chronic obstructive pulmonary disease, and neurodegenerative disease (dementia and parkinsonism) was assessed by interview and verified by reviewing medical records [14–17].

### Statistical analyses

We used the independent samples t-test (for continuous variables with a normal distribution), the Wilcoxon signed-rank test (for continuous variables with a skewed distribution), or the Chi squared test (for categorical variables) to investigate differences in characteristics between participants with pathology-confirmed diagnoses (certain cancer) and those with non pathology-confirmed diagnoses (probable and possible cancer). Furthermore, we compared cancer site specific percentages. An overview of the different ICD-10 codes used for categorisation into different cancer sites is presented in Supplementary Table 1.

Next, we explored a potential trend of pathological confirmation of cancer diagnoses over the years by plotting the number of incident pathology-confirmed and non pathology-confirmed diagnoses per calendar year. We tested the association between year of diagnosis and source of diagnosis (with or without pathological confirmation) formally using logistic regression models. This analysis was performed for all cancer sites combined and for the five most frequently non pathology-confirmed cancer sites separately. We constructed two nested models: model I was unadjusted; model II was adjusted for age at diagnosis (continuous).

We used two different methods to estimate overall survival. First, time to event was defined as follow-up time starting from date of diagnosis until date of death or date of censoring (loss to follow-up or end of the study period [January 1st, 2014]), whichever came first). Second, differences in overall survival between participants with and without pathological confirmation of the diagnosis were visualised by Kaplan–Meier curves and tested with a log-rank test. We additionally computed standardised survival curves to remove the influence of different distributions in age at diagnosis and sex between the groups [18, 19].

Standardised survival curves were created using a pooled logistic regression model for death including the following covariates: time (years), time squared (years), pathological confirmation of the diagnosis, age at diagnosis (continuous), and sex. Interactions between time and time squared with source of diagnosis were added to the model to allow for a flexible estimation of the baseline hazard. After fitting the pooled logistic model, we estimated the probability of death if all participants with cancer had a pathology-confirmed diagnosis, and the probability of death if all participants with cancer had a non pathology-confirmed diagnosis at each time point. Subsequently we calculated the difference in survival probability at each time point by taking the cumulative product as with Kaplan–Meier method. Confidence intervals (CIs) were obtained by bootstrapping. In sensitivity analyses, we repeated the analyses for cancer-specific survival and explored effect modification by median age, sex, education, and marital status.

Statistical analyses were performed using IBM SPSS Statistics Version 24.0 [20] and the packages ‘survival’ [21] and ‘survminer’ [22] from R software Version 3.3.2.

## Results

During a median (interquartile range) follow-up of 10.7 (6.3–15.9) years, 2698 out of 14,024 participants were diagnosed with cancer. The majority had a pathology-confirmed diagnosis (n = 2382 [88.3%]). Of the participants with a non pathology-confirmed diagnosis, 257 (9.5%) had a probable diagnosis and 59 (2.2%) had a possible diagnosis.

Characteristics of participants categorised into three groups, i.e., without cancer, with pathology-confirmed diagnosis, and with non pathology-confirmed diagnosis, are presented in Table 1. Participants with non pathology-confirmed diagnoses were older at diagnosis compared to participants with pathology-confirmed diagnoses (median age of 83.2 vs. 74.2 years, *P* < .001). Furthermore, they were more often women (55.7% vs. 47.6%, *P* = .007), lived more often without a partner (37.3% vs. 25.1%, *P* < .001), and had lower educational levels (*P* = .002). Lastly, participants with non pathology-confirmed diagnoses had more often hypertension (71.8% vs. 49.8%, *P* < .001) and more frequently a history of stroke (12.0% vs. 6.9%, *P* = .001), coronary heart disease (15.8% vs. 12.7%, *P* < .001), chronic obstructive pulmonary disease (19.0% vs. 13.7%, *P* = .011), and neurodegenerative disease (30.4% vs. 14.8%, *P* < .001) at diagnosis than participants with pathology-confirmed diagnoses.

**Table 1:** Characteristics of study population stratified by cancer diagnosis

Characteristic	Participants without cancer (N = 11,326)	Participants with cancer (N = 2698)		
		Pathology-confirmed diagnosis (N = 2382)	Non pathology-confirmed diagnosis (N = 316)	*P* value*
Age, years, median (IQR)	62.4 (57.7–72.7)	65.0 (60.2–72.0)	72.0 (66.1–78.1)	< .001
Sex, women, no. (%)	6912 (61.0)	1135 (47.6)	176 (55.7)	.007
Marital status, no. (%)				< .001
Living with partner	7418 (65.5)	1723 (72.3)	181 (57.3)	
Living without partner	3036 (26.8)	597 (25.1)	118 (37.3)	
Educational level, no. (%)				.002
Primary	2081 (18.4)	423 (17.8)	76 (24.1)	
Lower	4393 (38.8)	948 (39.8)	131 (41.5)	
Intermediate	2886 (25.5)	700 (29.4)	82 (25.9)	
Higher	1718 (15.2)	283 (11.9)	20 (6.3)	
Body mass index, kg/m^2^, mean (SD)	26.9 (4.2)	26.7 (3.8)	26.3 (3.7)	.137
Smoking, no. (%)				.001
Current	2313 (20.4)	608 (25.5)	90 (28.5)	
Former	4950 (43.7)	1106 (46.4)	109 (34.5)	
Never	3838 (33.9)	629 (26.4)	104 (32.9)	
Alcohol use, no. (%)	7843 (69.2)	1683 (70.7)	188 (59.5)	.287
Age at cancer diagnosis, years, no. (%)				
45–65		372 (15.6)	8 (2.5)	
65–75		897 (37.7)	42 (13.3)	
75–85		870 (36.5)	136 (43.0)	
> 85		243 (10.2)	130 (41.1)	
Median (IQR)		74.2 (68.0–80.3)	83.2 (78.0–88.0)	< .001
Comorbidities at cancer diagnosis, no. (%)				
Stroke		164 (6.9)	38 (12.0)	.001
Coronary heart disease		302 (12.7)	50 (15.8)	< .001
Hypertension		1186 (49.8)	227 (71.8)	< .001
Diabetes		324 (13.6)	37 (11.7)	< .001
Chronic obstructive pulmonary disease		326 (13.7)	60 (19.0)	.011
Neurodegenerative disease		353 (14.8)	44 (30.4)	< .001

Characteristics are measured at entry in the Rotterdam Study except for age at cancer diagnosis and comorbidities. Missing values are not imputed and therefore numbers do not always sum up to 100%

*IQR* interquartile range, *N* number of participants, *SD* standard deviation

*Two sided *P* values were calculated using the independent samples t-test (for continuous variables with a normal distribution), the Wilcoxon signed-rank test (for continuous variables with a skewed distribution), or the Chi squared test (for categorical variables) to investigate differences in characteristics between participants with pathology-confirmed diagnoses and participants with non pathology-confirmed diagnoses

Most frequently diagnosed cancer sites that were non pathology-confirmed included central nervous system (66.7% of all central nervous system cancers), hepato-pancreato-biliary (44.5%), unknown primary origin (31.2%), lung and mesothelioma (19.7%), and urinary tract (17.5%, Table 2). There was no statistically significant relation between pathological confirmation of these cancer sites with calendar year after adjustment for age at diagnosis, indicating that the number of participants with a pathology-confirmed diagnosis did not increase or decrease during the study period (Supplementary Fig. 1 and Table 3).

**Table 2:** Overview of number of pathological confirmations per cancer type

Cancer site	Pathology-confirmed diagnosis (N = 2382)	Non pathology-confirmed diagnosis (N = 316)	All cancer diagnoses (N = 2698)
Head and neck	83 (94.3)	5 (5.7)	88
Oesophagus and gastric	140 (97.9)	3 (2.1)	143
Colorectal	397 (96.6)	14 (3.4)	411
Hepato-pancreato-biliary	81 (55.5)	65 (44.5)	146
Lung and mesothelioma	314 (80.3)	77 (19.7)	391
Bone and soft tissue	19 (90.5)	2 (9.5)	21
Breast	341 (96.6)	12 (3.4)	353
Female genital organs	112 (94.9)	6 (5.1)	118
Male genital organs	387 (95.6)	18 (4.4)	405
Unitary tract	165 (82.5)	35 (17.5)	200
Central nervous system	9 (33.3)	18 (66.7)	27
Haematological	188 (90.0)	21 (10.0)	209
Other	80 (88.9)	10 (11.1)	90
Unknown primary origin	66 (68.8)	30 (31.2)	96

Numbers are displayed in total number (percentage per row)

**Table 3:** Association between calendar year of diagnosis and pathological confirmation of the cancer

Cancer site	N pathology-confirmed diagnosis	N non pathology-confirmed diagnosis	Model I		Model II	
			OR (95% CI)	*P*	OR (95% CI)	*P*
All cancer sites	2382	316	1.01 (1.00–1.03)	0.13	1.01 (0.99–1.03)	0.27
Hepato-pancreato-biliary	81	65	0.97 (0.92–1.02)	0.25	0.95 (0.89–1.02)	0.15
Lung and mesothelioma	314	77	1.05 (1.01–1.09)	0.02	1.03 (0.99–1.08)	0.12
Unitary tract	165	35	0.96 (0.91–1.02)	0.21	0.96 (0.90–1.03)	0.26
Central nervous system	9	18	1.05 (0.93–1.19)	0.45	1.06 (0.94–1.21)	0.36
Unknown primary	66	30	1.06 (0.99–1.14)	0.10	1.05 (0.98–1.13)	0.17

*CI* confidence interval, *N* number of participants, *OR* odds ratio

Model I = unadjusted. Model II = adjusted for age at diagnosis (continuous)

**Fig. 1:**
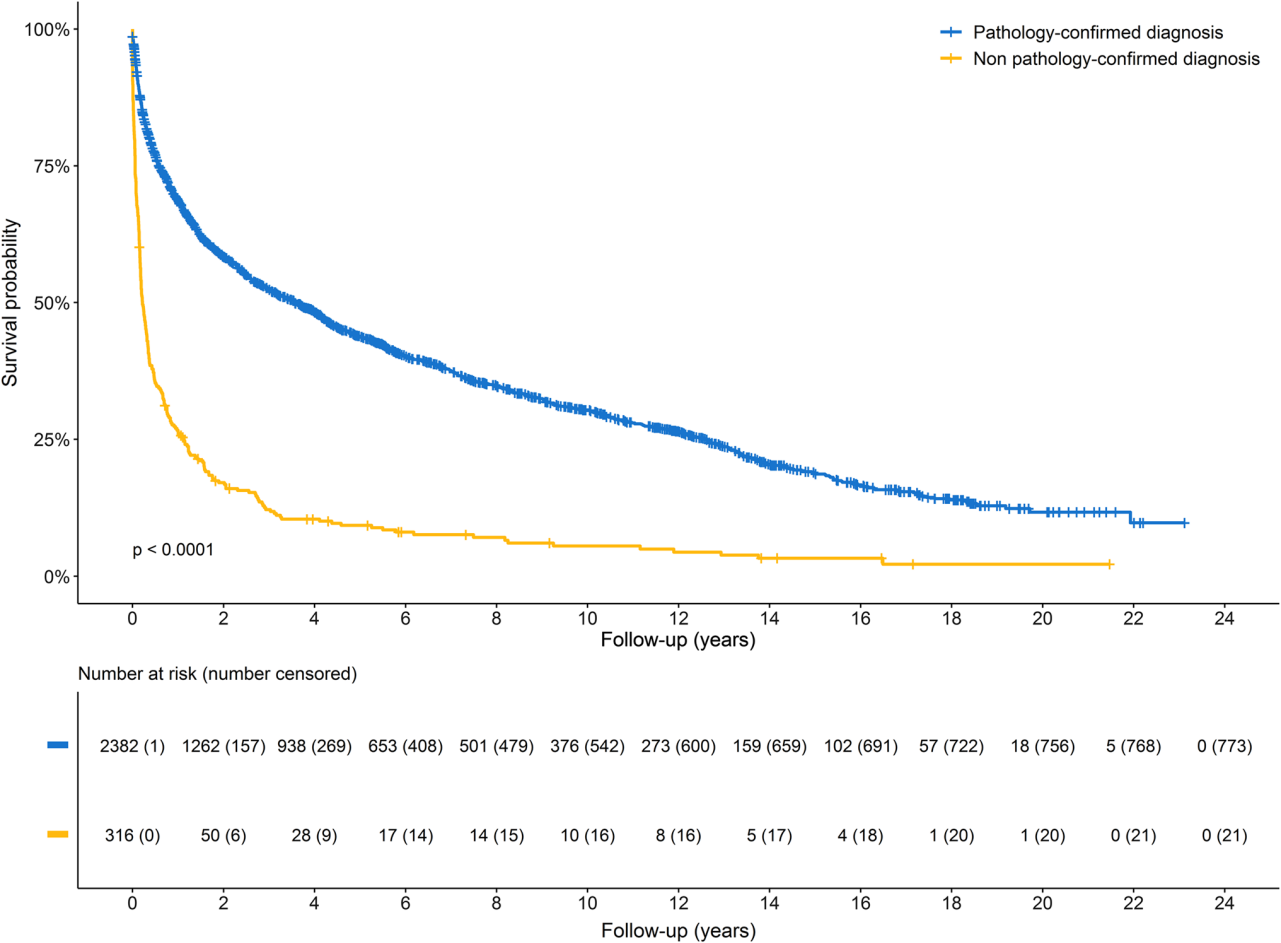
Kaplan–Meier curves for overall survival of participants with a pathology-confirmed diagnosis (blue) or a non pathology-confirmed diagnosis (yellow). Participants with a non pathology-confirmed diagnosis had significantly worse overall survival compared to those with a pathology-confirmed diagnosis (*P* of log-rank test < 0.0001). Participants were censored if they if they were lost to follow-up or at the end of the study period (January 1st, 2014), whichever came first

**Fig. 2:**
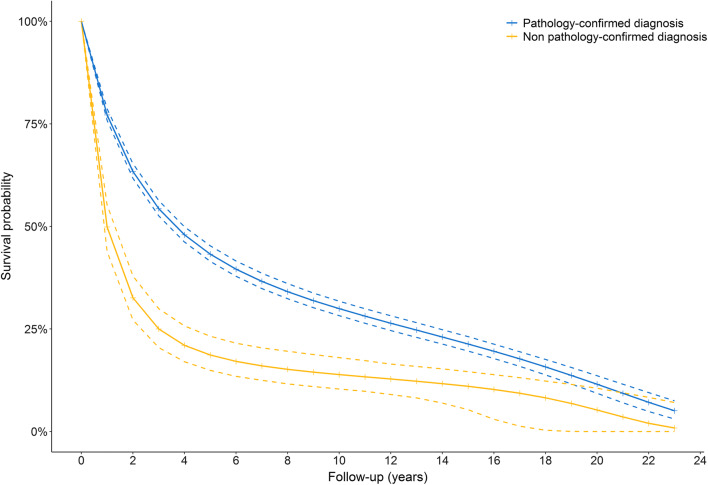
Standardised survival curves of individuals with a pathology-confirmed diagnosis (blue) or a non pathology-confirmed diagnosis (yellow). Dashed lines represent 95% confidence intervals. Survival curves are adjusted for age at diagnosis and sex. The risk difference of overall survival between participants with a non pathology-confirmed and a pathology-confirmed diagnosis is 30.8% after 1 year, 29.3% after 2 years, and 22.5% after 5 years

Of the 2382 participants with a pathology-confirmed diagnosis, 1154 participants (48.4%) died from cancer and 455 participants (19.1%) died due to other causes, such as heart failure, dementia, and cardiac arrest. Among participants with non pathology-confirmed diagnoses, 231 (73.1%) died from cancer, and 63 participants (19.9%) died from other causes. The overall survival of participants with non pathology-confirmed diagnoses was lower compared to participants with pathology-confirmed diagnoses (*P* for log-rank test < 0.0001, Fig. 1). After adjusting for age at diagnosis and sex, the overall survival in participants with non pathology-confirmed diagnosis was 30.8% (95% CI 25.2%; 36.2%) lower 1 year after diagnosis compared to participants with pathology-confirmed diagnoses (survival probability was 32.6% vs. 63.4%, respectively, Fig. 2). Two and five years after diagnosis, the difference in survival was 29.3% (95% CI 24.2%; 33.9%) and 22.5% (95% CI 17.7%; 26.4%), respectively. Cancer-specific survival probability was comparable to overall survival probability, with a lower cancer-specific survival in participants with non pathology-confirmed diagnoses than in participants with pathology-confirmed diagnoses (37.2% vs. 67.4%, respectively, Supplementary Fig. 2). No significant effect modification was observed across different strata of median age, sex, education, and marital status.

## Discussion

In a large population-based cohort study, we showed that non pathology-confirmed diagnoses of cancer represent an additional ten percent of cancer diagnoses besides pathology-confirmed diagnoses. Pathological confirmation of cancer was associated with multiple participant characteristics, comorbidities, cancer site, and survival. The proportion of participants with pathology-confirmed diagnoses did not change over time.

In line with previous studies investigating characteristics of patients with unstaged cancer [4–9], we found that participants with a non pathology-confirmed diagnosis were on average older compared to those with a pathology-confirmed diagnosis. There are different reasons for this observation. First, older patients have more comorbidities that may be of greater health concern than a potential cancer diagnosis [23]. Therefore, the diagnostic cancer work-up may be partly omitted. Furthermore, older patients are sometimes more vulnerable, limiting the ability to obtain pathology material for diagnosis through invasive procedures, such as endoscopic retrograde cholangiopancreatography (ERCP) for pancreatic cancer. In addition, age and comorbidities are associated with potentially less intensive treatment assignment including palliative radiotherapy and hormonal therapy [24, 25]. Although pathological confirmation of the cancer is often preferred, it may not always be mandatory for these treatment regimens [23, 26].

Lack of therapeutic consequences of pathological confirmation may explain why cancers with a poor survival in particular, such as cancer of central nervous system, hepato-pancreato-biliary tract, and lung were often diagnosed without pathological confirmation. Cancers at these organ sites are often detected in a more advanced stage, limiting treatment options to palliative treatments. Furthermore, we found that participants with cancer of unknown primary origin often had no pathological confirmation, suggesting that these participants had metastasised disease and did not undergo further diagnostic testing [27]. Another explanation for this finding is that cancers at these sites are less accessible for obtaining tumour tissue, in particular regarding cancers of the central nervous system. Lastly, cancers in the urinary tract including renal cell carcinoma and prostate cancer were often non pathology-confirmed. These cancers are often diagnosed non-invasively with imaging modalities (renal cell carcinoma) or by the assessment of tumour markers (prostate cancer). Watchful waiting is increasingly being considered as an option for older, vulnerable patients with regard to prostate cancer [28], resulting in a lower number of pathology-confirmed diagnoses.

Interestingly, we showed that participants with a non pathology-confirmed diagnosis of cancer had worse overall and cancer-specific survival compared to participants with a pathology-confirmed diagnosis. Although the number of cancers with a poor survival was more frequently represented among non pathology-confirmed diagnoses, this difference in cancer type distribution cannot completely explain the observed difference in survival. Therefore, the difference in survival may indicate that pathological confirmation is more often omitted in patients with a ‘worse’ cancer prognosis. In contrast, previous studies found a better survival in patients with unstaged cancer. For instance, unstaged colorectal cancer was associated with higher survival compared to patients with distant-staged cancer [5]. Furthermore, non pathology-confirmed early stage lung cancer patients had a better cancer-specific survival compared to patients with a pathology-confirmed diagnosis, due to the occurrence of benign lung nodules among the diagnosed cancers without pathological confirmation [29]. This misclassification of benign tumours may partly explain the discrepancy in survival between previous studies and the current study. Although we cannot exclude that we also classified benign tumours as non pathology-confirmed cancers, the number of misclassified tumours is expected to be low because of the persistent poor cancer-specific survival of participants with a non pathology-confirmed diagnosis.

We previously showed that cancer registries primarily rely on pathology databases as signalling source of cancer diagnoses, resulting in under-registration of non pathology-confirmed diagnoses [30]. The findings of our current study indicate that under-registration of such cancers may result in underestimation of the cancer incidence, and in overestimation of cancer survival. Furthermore, non pathology-confirmed diagnoses were related to multiple characteristics including age, sex, smoking status, and education, and to cancer site. Most aetiological studies only include patients with a pathology-confirmed diagnosis, which may induce information bias and result in inaccurate estimates of association [31]. For these reasons, our results suggest that registries and research studies should also include patients with non pathology-confirmed diagnoses for potential sensitivity analyses.

The main strength of this study is the unique setting of the Rotterdam Study in which cancer registration relies on medical letters and medical records from the general practitioners in addition to signalling of diagnoses through the nationwide pathology database as well as linkage to the national cancer registry. This allowed us to investigate also non pathology-confirmed diagnoses not registered through the pathology database. Furthermore, we estimated survival by computing standardised survival curves in addition to the unadjusted Kaplan–Meier curves. Unfortunately, we could not adjust these survival curves for frailty. Although the Rotterdam Study started to collect data on frailty from 2009 onwards, including weight loss, physical activity, weakness, slowness, and fatigue to calculate the Fried frailty index [32], this was not available for the majority of the participants (< 20%), or—if available—was measured several years after cancer diagnosis. Another limitation is that the date of diagnosis is determined differently for non pathology-confirmed and pathology-confirmed diagnoses. It is plausible that participants with non pathology-confirmed diagnoses were diagnosed sooner, resulting in a slightly longer cancer-specific survival. Lastly, we cannot rule out that non pathology-confirmed diagnoses are benign tumours. However, we classified cancers based on all the available information from medical letters and medical records, limiting the number of false positive diagnoses. In addition, we showed that participants with non pathology-confirmed diagnoses had worse cancer-specific survival persistent over time, suggesting that these cancers were malignant.

In conclusion, we show that purely non pathology-confirmed diagnoses represent ten percent of the total number of diagnosed cancers, besides pathology-confirmed diagnoses. Pathological confirmation is associated with several characteristics and with worse overall and cancer-specific survival. Our findings suggest that missing data or exclusion of non pathology-confirmed diagnoses may result in underestimation of the true cancer incidence, overestimation of survival, and potentially may bias aetiological research findings.

## Electronic supplementary material

Below is the link to the electronic supplementary material.

Supplementary material 1 (DOCX 273 kb)
