# Supplementary material for: Pathology-confirmed versus non pathology-confirmed cancer diagnoses: incidence, participant characteristics, and survival
Source: Eur J Epidemiol. 2019 Dec 20;35(6):557–65. doi: 10.1007/s10654-019-00592-5 (PMC7320936; doi:10.1007/s10654-019-00592-5)
Supplement: Supplementary file 1 — Supplementary material 1 (DOCX 273 kb) [file 10654_2019_592_MOESM1_ESM.docx]

**Pathology-confirmed versus non pathology-confirmed cancer diagnoses: incidence, participant characteristics, and survival**

Kimberly D. van der Willik, MD^a,b^, Liliana P. Rojas-Saunero, MD^b^, Jeremy A. Labrecque, PhD^b^, M. Arfan Ikram, MD PhD^b^, Sanne B. Schagen, PhD^a,c^, Bruno H. Stricker, MD PhD^b^, Rikje Ruiter, MD PhD^b^

**Affiliations**

^a^ Department of Psychosocial Research and Epidemiology, Netherlands Cancer Institute, Plesmanlaan 121, 1066 CX Amsterdam, the Netherlands

^b^ Department of Epidemiology, Erasmus MC - University Medical Center Rotterdam, PO Box 2040, 3000 CA Rotterdam, the Netherlands

^c^ Brain and Cognition, Department of Psychology, University of Amsterdam, Nieuwe Achtergracht 129B, 1018 WT Amsterdam, the Netherlands

**Corresponding author**

Rikje Ruiter, MD PhD. Department of Epidemiology, Erasmus MC – University Medical Center Rotterdam, PO Box 2040, 3000 CA Rotterdam, the Netherlands. Tel: +31107044294. E-mail address: [r.ruiter@erasmusmc.nl](mailto:r.ruiter@erasmusmc.nl).


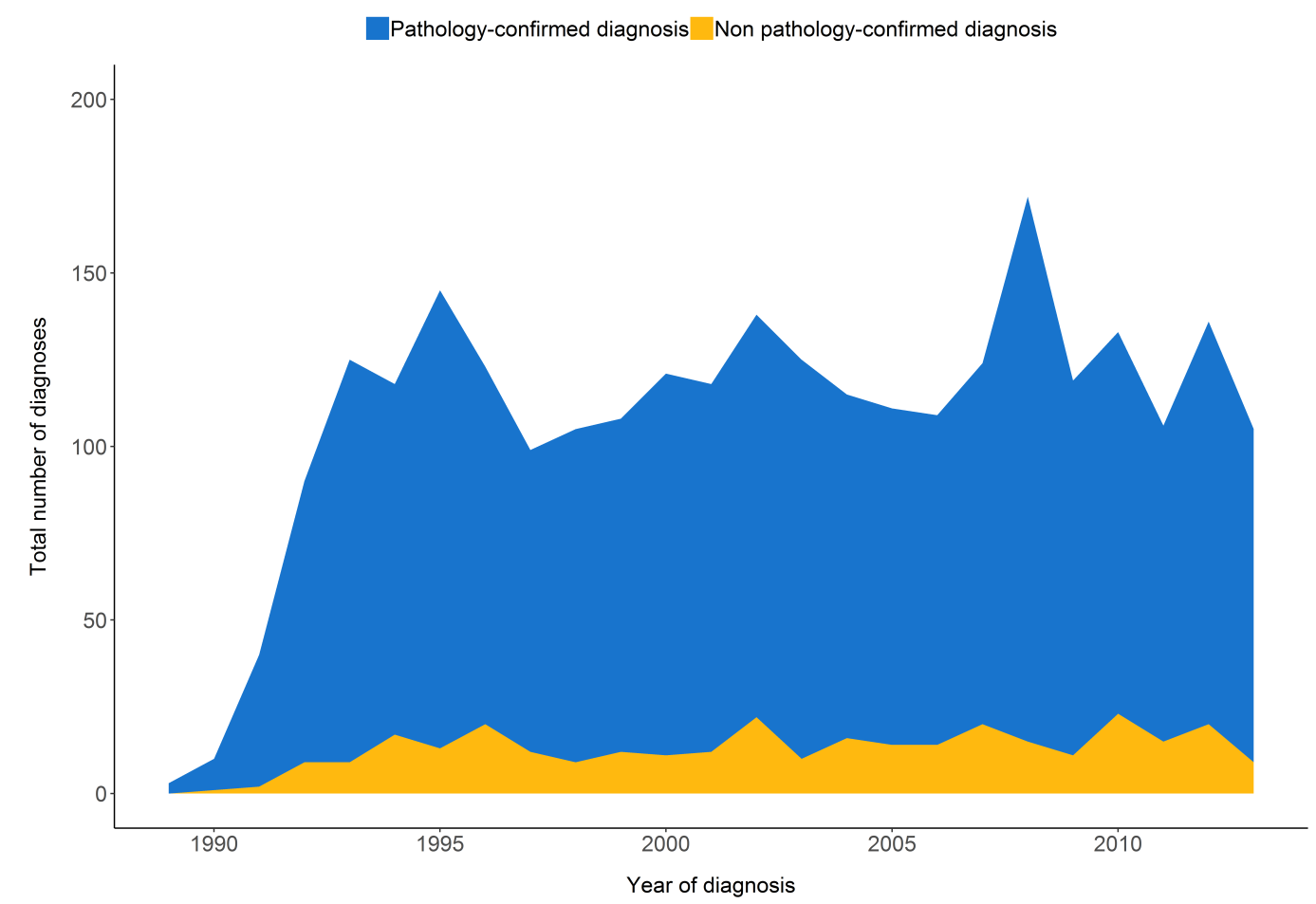


**Supplementary Fig. 1** Trends in cancer diagnoses per calendar year of cancer diagnosis. Number of pathology-confirmed diagnoses (blue) and non pathology-confirmed diagnoses (yellow) are shown as the total number of cancer diagnoses.

**
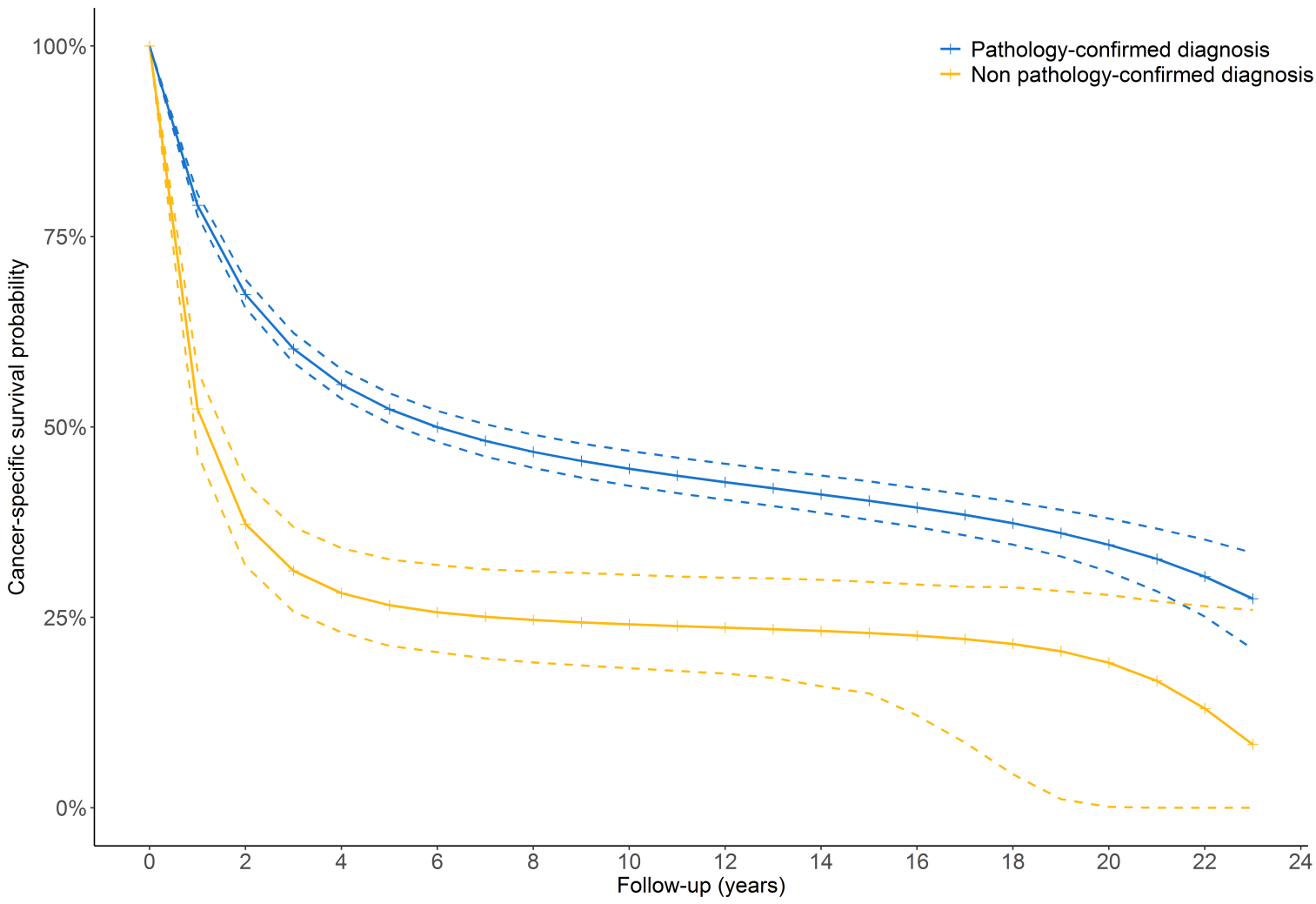
**

**Supplementary Fig. 2** Standardized survival curves of individuals with a pathology-confirmed diagnosis (blue) or a non pathology-confirmed diagnosis (yellow). Dashed lines represent 95% confidence intervals. Survival curves are adjusted for age at diagnosis and sex. The risk difference of cancer-specific survival between participants with a non pathology-confirmed and a pathology-confirmed diagnosis is 30.2% after one year, 29.1% after two years, and 24.3% after five years.

**Supplementary Table 1** Overview of the used ICD-10 codes per cancer site.

| **Organ system** | **Corresponding ICD-10 code** |
| --- | --- |
| Head and neck | C01-C14,C30,C32,C69,C73 |
| Esophagus and gastric | C15,C16 |
| Colorectal | C18-C20 |
| Hepato-Pancreato-Biliary | C22-C25 |
| Lung and mesothelioma | C34,C45 |
| Bone and soft tissue | C40,C41,C49 |
| Breast | C50 |
| Female genital organs | C51-C57 |
| Male genital organs | C60-C63 |
| Unitary tract | C64-C68 |
| Central nervous system | C70-C72 |
| Hematological | C81-C96 |
| Other | C17,C21,C26,C37-C39,C43,C48,C75,C76 |
| Unknown primary origin | C80 |

ICD = International Classification of Diseases.
